# Special Care Dentistry in a Patient with Prader–Willi Syndrome through the Use of Atraumatic Restorative Treatment under General Anesthesia

**DOI:** 10.1155/2017/7075328

**Published:** 2017-11-28

**Authors:** Caio Vinícius Gonçalves Roman-Torres, Sérgio Takashi Kussaba, Yasmin Comoti Vita Bantim, Roberta de Barros Antunes Almeida de Oliveira

**Affiliations:** ^1^Department of Dentistry, University of Santo Amaro, Santo Amaro, SP, Brazil; ^2^Cathedral College, Boa Vista, RR, Brazil; ^3^Pediatric Dentistry, Boa Vista, RR, Brazil

## Abstract

Prader–Willi syndrome described in 1956 has a genetic origin, affecting both genders, varying in presence and intensity from individual to individual. A precocious diagnosis, before the manifestation of symptoms, has brought some improvement in the quality of life of the carriers in the last years. The objective of this case report was to describe the treatment realized in a 3-year-old boy who presented grade II obesity, difficulty of locomotion, hypotonia, and history of cardiopathy. A dental treatment under general anesthesia was defined, allowing an oral adequation in a single section, in which it was planned the extraction of the element 74 and atraumatic restorative treatment (ART) technique in the other teeth. The precocious intervention in this 3-year-old patient by the therapy realized with ART under general anesthesia was done with success, avoiding unnecessary extractions, preserving dental elements, and maintaining the oral cavity in adequate function.

## 1. Introduction

Prader–Willi syndrome (PWS) is a systemic disease and occurs with an incidence of 1/10,000–1/30,000 [1], described by Prader, Labhart, and Willi in 1956; it is considered a neurobehavioral disease currently pointed as one of the most frequent causes of chromosomal microdeletions [2], and it is associated with endocrine and behavioral disturbances that may determine repercussions in oral cavity [3]. Although the diagnosis may be easily proven on the basis of well-defined clinical criteria, even in the neonatal period, the increasing availability and application of molecular techniques indicate that genetic tests are needed to confirm the diagnosis [4].

The precocious clinical manifestations of PWS are unspecific and may be confused with others of the nervous system [5]. The almond-shaped eyes with upslanting palpebral fissures and triangular mouth are orofacial characteristics described for PWS patients [6] that also include infant lethargy and hypotonia, causing poor nutrition and growth deficit; intellectual development disturbance and hypogonadism; hyperphagia, leading to morbid obesity if not controlled; short stature, *habitus corporeo*; and a typical behavioral phenotype that includes temper tantrums and compulsive traits [7].

A study with PWS patients demonstrated that all of them presented, at least, five of the following typical facial characteristics: prominent forehead, almond-shaped eyes, downturned mouth, narrow face, thin upper lip, micrognathia, and small-appearing mouth [5]. The retrospective analysis of 35 PWS cases in Brazil evinced that hypotonia was present in the first year of life of all patients, leading to nutrition problems in the great majority of the cases [8].

The presence of a precarious oral hygiene encounters PWS patient's systemic conditions due to the cognitive and self-care deficit associated with the patient's behavioral disturbance, evinced by a prior poor oral hygiene. Besides, it is suggested that the lack of precocious multidisciplinary support may contribute to the opportunistic disease advance [9].

A poor oral hygiene as well as a low saliva production and viscosity were observed in a 13-year-old PWS patient's analysis. The enamel demineralization was observed; epithelial alterations on the tongue, presence of candidiasis, and small hands make oral hygiene maintenance difficult [10].

Emotional disturbances predispose PWS carriers to the compulsive act of biting their oral mucosa and bruxism, resulting in dental wear [11]. Studies demonstrate that PWS patients may present dental caries [3, 10, 11], enamel hypoplasia [10], low salivary flow [6, 10, 12], periodontal disease, incisal and occlusal wear [11], dental erosion [6], rampant caries, oral microsomia [10], reflow, malocclusion [3], candidiasis [10], and oral mucosa erythematous lesions [11].

The PWS needs to be well known and worked in the dental office through a multidisciplinary perspective, aiming at an accurate and effective assistance and determining a reestablishment of the patient's oral health [3]. The scientific literature mentions that there is a scarcity of studies about PWS patients' oral conditions [12, 13]; however, there are reports of ambulatory services, even with patients with aggressive behavior [11]. Other studies do not report intervention under general anesthesia [10, 14].

The reports of care in patients with PWS with non-ambulatory treatment indicate that all clinical treatment should be performed under general anesthesia to control infection and intraoral adequacy. Therefore, the objective of this work was to report a case of a PWS patient who was subjected to atraumatic restorative treatment (ART) under general anesthesia.

## 2. Case Report

A 3-year-old male of 28 kg was brought by the genitor to the Cathedral College Dental Clinic, Roraima, Brazil, and since the beginning, he offered resistance to conventional dental treatment. The parents received information verbally and in writing about the procedures performed in the conventional dental treatment and with ART. After the explanations, they opted for ART.

During the anamnesis, the mother reported that the child tends to exhibit mood swings, bouts of aggressiveness towards family, and also self-injurious behavior when contradicted. She also reported certain resistance by him to obey her commands during any procedure that he was subjected to. In the initial appointment, it was observed a good degree of understanding and a good level of socialization and interaction with the student who examined him at the school clinic. Nonetheless, signs of resistance to some verbal commands were noticed, making the initial clinical examination impossible. The mother strictly controls his feeding, providing a hypocaloric consumption diet, but because of the eating compulsion caused by the syndrome, the child's weight is increasing considerably. According to the mother's report, the patient is in constant endocrinological, neurological, and cardiac monitoring, two years ago, since the diagnosis of the syndrome. She reports the use of the sedative medication levomepromazina (Levozine®; Cristália) and O_2_ drops in the morning.

**Figure 1:**
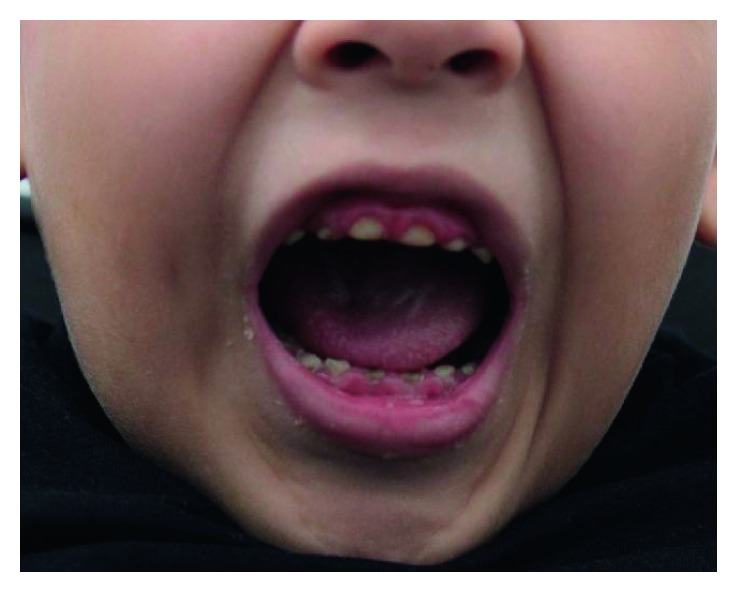
Gingival inflammation, dental abrasion, and psychological aspects that made it difficult to start treatment.

In the general physical examination, he presented grade II obesity, difficulty in locomotion, hypotonia, and a history of cardiopathy. After three clinical sessions for patient's conditioning, a discreet empathy was achieved with the service team who managed to perform the intraoral clinical examination. Multiple sources of oral infection were observed, such as dentoalveolar abscess, generalized enamel demineralization, presence of biofilm, atypical unobtrusive swallowing, hypotonic tongue, and predominantly oral breathing (Figure 1). Disturbances in the eruption sequence or lack of the teeth were not evinced. After frustrating attempts to execute additional examination of periapical radiographs, it was defined, along with the pediatric dentistry clinic, a dental treatment under general anesthesia, allowing an oral environment adequation in a single session, being planned dental extraction of element 74 with extensive caries lesion, and atraumatic restorative technique (ART) in the elements: 55, 54, 52, 51, 61, 62, 64, 65, 75, 73, 72, 71, 81, 82, 83, 84, and 85 as well as dental scaling and root planing. For this purpose, it was requested surgical risk and preanesthesia evaluation for elective procedure in surgical center. The ART technique, the removal of the decayed tissue, the insertion of the glass ionomer cement (GIC), and dental scaling and root planing (DSRP) were applied under general anesthesia.

**Figure 2:**
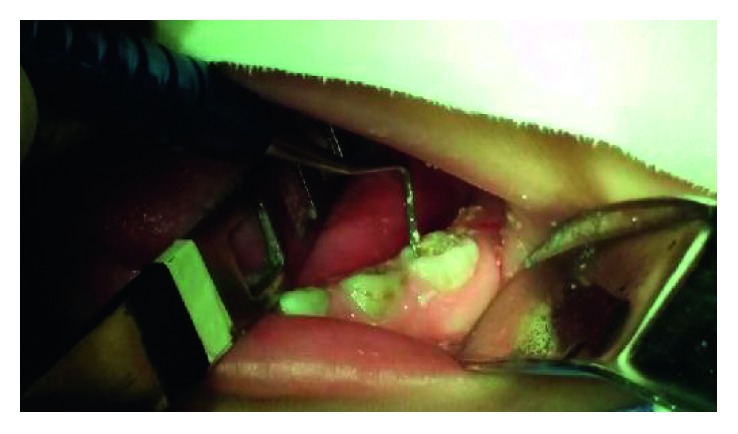
The removal of decayed tissue with ART.

**Figure 3:**
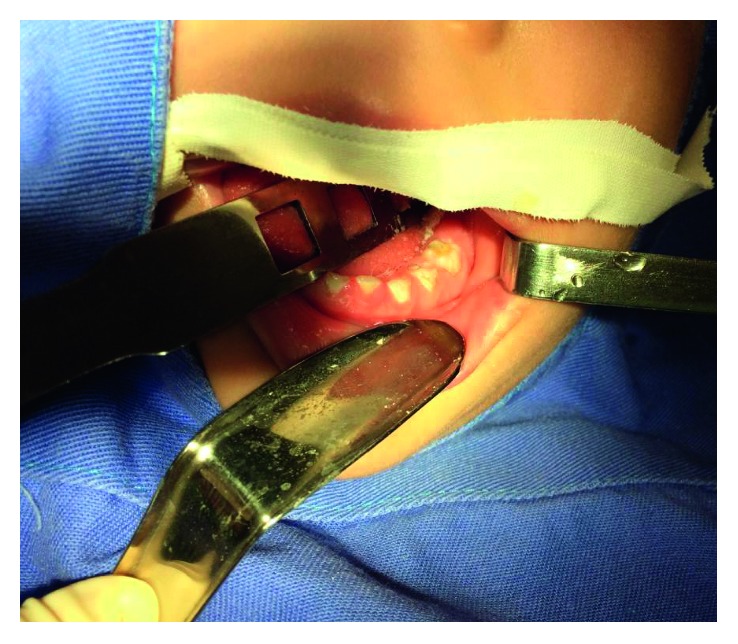
The cavity restored with the glass ionomer restorer.

For the removal of decayed tissue with ART (Figure 2), the restorer kit (Cyann ART Duflex®; SS White) was used, removing the necrotic dentin until the exposure of the secondary dentin. In the end of the cleaning, cavity conditioning was performed with polyacrylic acid (Vitro Conditioner®; DFL) on the smear layer, being thoroughly washed with sterile water and dried with sterile cotton. The cavity was restored with the glass ionomer restorer (Ketac Molar™ ESPE-3M) and the excess material was firmly pressed into the cavity and enamel adjacent fissures with a lubricated glove finger and sealed with fluoride varnish (Fluorniz®; SS White) (Figure 3).

A local anesthesia was applied in the lower left molar region with 2.0% of lidocaine hydrochloride with epinephrine 1 : 100,000 (Alphacaine®; New DFL) for hemostasis and postoperative analgesia control followed by extraction of the element 74 and suture with absorbable thread of polyglactin 910 4-0 (Vicryl™; Ethicon®) with simple stitches interrupted for maintenance of blood clot in the alveolus, because it is a premature extraction, once it is necessary, as long as there is still alveolus.

## 3. Discussion

The PWS patient demands that all the family system be committed; psychosocial support is compulsory due to the multiple challenges of living with and taking care of a child/young adult with PWS [15]. Thus, caregivers and family members are informed about the necessity of psychological care for the family. The aspects of PWS observed in the literature are compatible with the reported case where aggression and negative suggestibility were observed and which are common in these patients. Behavioral and psychiatric issues interfere more in the quality of life in PWS adult patients [16].

The neurological development is achieved on average twice the normal age, with the ability of speech beginning at 2 years old [7], with delayed motor and language development and poor diction. Grade II obesity, difficulty of locomotion, generalized hypotonia, and history of cardiopathy are clinical signs observed in the patient, being compatible with the information noted in literature [1, 4]; there is a need to control feeding [6] which is strictly accomplished by the mother, providing hypocaloric consumption diet. However, on the basis of the syndrome and eating compulsion, weight is considerably increasing.

The PWS patient presents with neurobehavioral alterations of extreme interest to the dentistry field. For this reason, the development of strategies that promote general and dental health for them should be the goal of the dental team that is involved with its care [3]. This case presented attempt and approximation between the patient and the professional with discreet empathy. However, the construction of this relation was insufficient, leading the case to an intervention under general anesthesia in search of a necessary control of the oral infection.

Patients who cooperate with dentists can be treated using local anesthesia, including orthodontic treatment. The complementary radiographic exams subsidize the clinic providing reliable information that is not always possible to be noticed in the clinical examination. The treatment towards uncooperative patients is more complex. The sedation levels, respiratory depression, and possible adverse pharmacological effects of oral sedation are difficult to control in these vulnerable patients. When the behavioral techniques fail, long and invasive treatments and more critical procedures may be performed under general anesthesia [6]. Unfortunately, for a distance of 500 miles, there is no suitable place to care for these patients properly. Besides the necessary displacement, there is a waiting period of some months for the first consultation to be made.

The oral changes observed are compatible with those present in the scientific literature with multiple dental caries [3, 10, 11], tooth 74 with pulp necrosis and dentoalveolar abscess, generalized enamel demineralization [10], reasonable oral hygiene [9, 10, 14], presence of biofilm [12], atypical unobtrusive swallowing, hypotonic tongue, and predominantly oral breathing.

The approach based on ART enables cost savings [17]; it is promptly accepted by children and results in the maintenance of many teeth that could be extracted depending on the circumstances [18]. The analysis of ART protocols when compared with the conventional restoration treatment in the clinic and under general anesthesia had indicated that ART seems to be a more efficient protocol for the treatment of patients with restrictive disability, many of whom have difficulty to cope with the conventional restoration treatment [19].

The precocious diagnosis and complete orofacial examination are important to optimize the planning and management of the treatment and to minimize the risk of progression of the symptoms in development. Once the diagnosis of the syndrome is confirmed, dental appointments should be initiated in a collaborative work with parents to inform about the possible dental problems and instructions of how to avoid them. The early introduction of healthy nutrition and oral hygiene practices and the use of fluoride supplements, when appropriate, would help to avoid deleterious dental consequences [20].

The planned intervention in this 3-year-old patient by means of intraoral infection control therapy in a single session under general anesthesia and appropriate postoperative accompaniment with the pediatric dentistry clinic for monitoring and conditioning of this patient to ambulatory interventions avoided unnecessary extractions, preserving dental elements that are essential to maintain an adequate quality of life.

## 4. Conclusion

The present study demonstrated the clinical intervention in a young patient with PWS when there is difficulty on conventional treatment. The choice of a hospital environment with general analgesia and ART application proved to be opportune and viable, ensuring the recovery of the patient's oral health quality in a shorter timely manner.
